# Postoperative Heart Failure with Preserved Ejection Fraction Induced by Flumazenil Administered for Remimazolam Antagonism

**DOI:** 10.1155/2022/8923008

**Published:** 2022-11-12

**Authors:** Keito Koh, Takeshi Omae, Sonoko Sakuraba, Masateru Kumemura, Sho Yamazaki, Hiroshi Yunoki

**Affiliations:** ^1^Department of Anesthesiology and Pain Medicine, Juntendo University Shizuoka Hospital, 1129 Nagaoka, Izunokini-Shi, Shizuoka 410-2211, Japan; ^2^Department of Anesthesiology, Nishijima Hospital, 2835-7 Ooka, Numazu-Shi, Shizuoka 410-0022, Japan

## Abstract

Remimazolam is an ultrashort-acting benzodiazepine intravenous anesthetic characterized by rapid awakening after anesthesia. However, the method for administering remimazolam in clinical practice remains unclear. Here, we report a case of postoperative heart failure with preserved ejection fraction (HFpEF) after antagonizing remimazolam with flumazenil. An 82-year-old woman was scheduled to undergo lumbar laminectomy for lumbar spinal canal stenosis. Preoperative echocardiography revealed normal left ventricular systolic function, left atrial enlargement, and impaired left ventricular diastolic function. General anesthesia was induced with 10 mg/kg/h remimazolam and maintained with 0.8 mg/kg/h remimazolam intraoperatively. Before extubation, a total of 1.0 mg of flumazenil was administered. After extubation, the patient developed pulmonary edema due to HFpEF. When remimazolam is administered in elderly patients with cardiac dysfunction, the maintenance dose should be customized according to the patient's general condition to minimize the dosage of flumazenil.

## 1. Introduction

Remimazolam is an ultrashort-acting benzodiazepine intravenous anesthetic that can be antagonized by flumazenil unlike propofol [1]. Although the administration of an antagonist is expected to result in more rapid awakening than propofol, many unanswered questions regarding its clinical use exist, including the appropriate maintenance dose for elderly patients and the timing of antagonist administration. Here, we report a case of postoperative heart failure (HF) with preserved ejection fraction (EF; HFpEF) after antagonizing remimazolam with flumazenil.

## 2. Case Presentation

**Figure 1:**
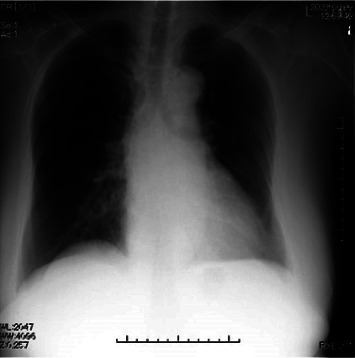
Preoperative chest radiograph.

An 82-year-old woman (height, 158 cm; weight, 68 kg) was scheduled to undergo laminectomy of three lumbar vertebrae for lumbar spinal stenosis in the prone position. She had undergone right femoral head replacement under general anesthesia 3 years before presentation. Comorbidities included hypertension and diabetes mellitus, which were treated with oral medications. Her blood pressure was maintained at 135/90 mmHg. The preoperative chest radiograph was no abnormality (Figure 1). Her preoperative electrocardiogram (heart rate, 86 beats/min) demonstrated a sinus rhythm. Echocardiography revealed a left ventricular EF (LVEF) of 66%, no asynergy, and left atrial (LA) enlargement with an LA diameter of 41 mm. The ratio of early to late diastolic transmitral flow velocity (E/A) was 1.40; her mitral annular velocity measured using tissue Doppler imaging showed an impaired relaxation pattern, and pulmonary venous flow demonstrated progression of the peak atrial systolic backward velocity. Based on the pseudo-normal pattern of E/A, LV diastolic dysfunction was diagnosed. She could perform daily activities independently, was classified as New York Heart Association Class I, and had an exercise tolerance level of 4 metabolic equivalents of tasks or higher.

**Figure 2:**
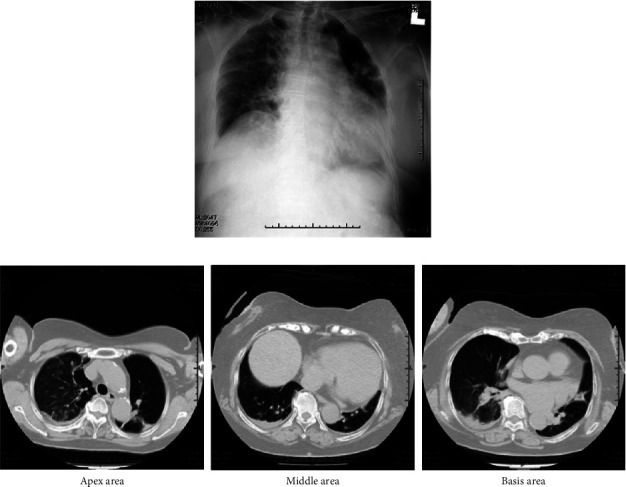
(a) Chest radiograph at the end of the surgery and (b) Chest computed tomography scan at the end of the surgery.

**Figure 3:**
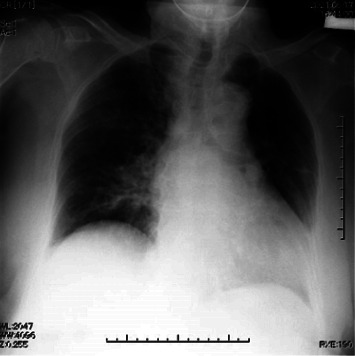
Chest radiograph at 4 hours after surgery.

**Figure 4:**
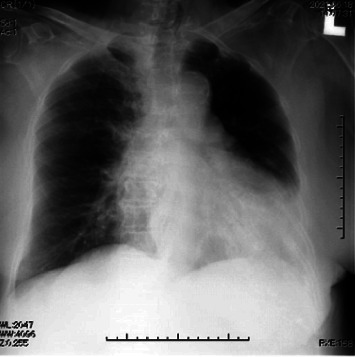
Chest radiograph on postoperative day 1.

**Figure 5:**
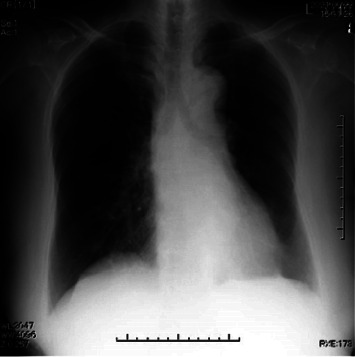
Chest radiograph on postoperative day 4.

General anesthesia was induced with remimazolam (10 mg/kg/h), remifentanil (0.5 *μ*g/kg/min), and rocuronium (50 mg) and maintained with remimazolam (0.8 mg/kg/h) and remifentanil (0.2 *μ*g/kg/min). No additional rocuronium was administered intraoperatively because of the use of a monitor-evoked potential. The bispectral index (BIS) value during anesthesia was 40–60. Fentanyl 200 *μ*g was administered 20 min before the end of surgery. The train of four ratios was 90%, with no residual muscle relaxants. Flumazenil (0.2 mg) was administered 5 min after surgery completion. However, she was not fully awake, with a BIS value of 60; therefore, an additional 0.2 mg of flumazenil was added. However, the patient was inadequately awake. Subsequently, flumazenil (0.2 mg) was administered every 5 min up to a total of 1.0 mg because the patient remained inadequately awake, with a BIS value of 70. The subsequent BIS value was 90, and the patient was able to follow instructions, indicating sufficient emergence. No sputum or upper airway obstruction was observed, and the patient was extubated. Her vital signs at the time of discharge from the operating room were as follows: blood pressure (BP), 200/100 mmHg; heart rate, 75 beats/min; O_2_ saturation, 92% (on 4 L of O_2_ administered via a mask); and no postoperative pain. The operative time was 1 h and 55 min, and the duration of anesthesia was 3 h and 14 min. The intraoperative infusion volume, blood loss volume, urine output, and intake/output fluid balance were 550 mL, 110 mL, 285 mL, and 1.1 mL/kg/h, respectively. Routine chest radiography performed 10 min after discharge from the operating room revealed pulmonary edema (Figure 2(a)). Simultaneously, computed tomography was performed (Figure 2(b)). Although she did not experience respiratory discomfort, the arterial gas analysis indicated hypoxemia with a partial arterial O_2_ pressure of 67.6 mmHg (on 3 L of O_2_ by mask) and a partial arterial carbon dioxide pressure of 51.5 mmHg. The amino-terminalpro-brain natriuretic peptide (NT-proBNP) level was 255 pg/mL, indicating mild HF. Antihypertensive and diuretic drugs were administered immediately. Chest radiography performed 4 hours after surgery revealed a slight improvement in pulmonary edema (Figure 3). The NT-proBNP level measured on postoperative day 1 remained almost unchanged at 231 pg/mL. Chest radiography demonstrated improvement in pulmonary edema (Figure 4). On postoperative day 4, the chest radiograph appeared normal, without an unelevated cardiothoracic ratio or pulmonary edema (Figure 5). One month postoperatively, the patient was discharged from the hospital, and her general condition was stable.

## 3. Discussion

In the present case, the patient developed postoperative pulmonary edema despite the absence of preoperative LV systolic dysfunction and excessive intraoperative fluid infusion. HF is generally classified according to LVEF as follows: HF with reduced EF (HFrEF, EF <40%); HF with midrange EF (40%≤ EF <50%), and HFpEF (EF ≥ 50%) [2]. The characteristic pathogenesis of HFpEF is acute pulmonary edema caused by a rapid increase in BP and LA pressure [3] in association with LV diastolic dysfunction [4]. Diagnostic criteria are as follows: (1) clinical symptoms of HF, (2) LVEF ≥50%, and (3) elevated LA pressure due to LV diastolic dysfunction. HFpEF is common in people aged ≥60 years, women, and patients with comorbidities, such as hypertension or diabetes mellitus [5]. HFpEF affects approximately half of HF patients [2]. However, diagnosing patients presenting with a few HF symptoms is difficult because LA pressure increases only on exertion [6]. As with HFrEF, HFpEF is primarily managed by symptomatic treatment, including fluid restriction and diuretic administration. Currently, no specific treatments for HFpEF exist [5]. Although echocardiography was not performed at onset, preoperative echocardiography revealed normal LV systolic performance and EF, LA enlargement, and LV diastolic dysfunction in our case. Furthermore, our patient developed postoperative pulmonary edema despite the absence of intraoperative fluid overload, postoperative pain, and upper airway obstruction. Based on these factors, the cardiologist diagnosed the pathogenesis of pulmonary edema as HFpEF. Our patient had several risk factors for HFpEF, including age >60 years, female sex, and comorbidities, including diabetes and hypertension. The patient did not exhibit typical HF symptoms, such as respiratory distress. Respiratory distress was not observed because of the patient's reduced activity level after surgery. The NT-proBNP level did not improve even after improvement in pulmonary edema, suggesting that the preoperative level might have originally been high, reflecting LV diastolic dysfunction.

Flumazenil exerts its antagonistic effect by competitively binding to central benzodiazepine receptors [7]. The most important cause of HFpEF in our case may be the sympathomimetic effect of the rapid emergence induced by flumazenil. Rapid emergence induced by flumazenil elevates serum norepinephrine and epinephrine, indicating that the sympathomimetic effect is increased [8]. Increased secretion of catecholamines increases systemic vascular resistance and elevates BP [8]. In our case, the flumazenil dosage was 1.0 mg, which was not extreme; however, the rapid increase in the afterload, which resulted from the elevation of BP induced by flumazenil, might have influenced the existing impaired LV diastolic function because of old age and hypertension. Subsequently, the circulatory regulatory system failed, resulting in a rapid increase in LA pressure, which might have led to HFpEF and pulmonary edema. The causal association between flumazenil and HF should be further studied in the future.

Remimazolam may cause a delayed awakening in elderly patients (age >65 years) at a maintenance dose of 0.8–1.0 mg/kg/h. The optimal maintenance dose in elderly patients is approximately half of that in nonelderly patients [9]. An intraoperative induction dose (1.0–1.2 mg/kg/h) and maintenance dose (0.1–0.2 mg/kg/h) of remimazolam can maintain a BIS value of 40–60 in patients aged >90 years [10]. The optimal maintenance dose of remimazolam in patients with American Society of Anesthesiologists physical status III is 0.56 mg/kg/h [11]. In our case, the induction and maintenance doses of remimazolam were 10 mg/kg/h and 0.8 mg/kg/h, respectively, administered in accordance with the package insert, and the intraoperative BIS value was 40–60; however, 1.0 mg of flumazenil was required for awakening. Although no detailed report has yet examined the reliability of BIS value for sedation levels with remimazolam, the algorithm for calculating BIS value is primarily adjusted for propofol, and the system with benzodiazepines is considered to be inferior [12]. Otherwise, remimazolam has been reported to show a higher BIS than propofol at the same sedation levels [13]. Hence, it seems appropriate to keep BIS levels higher when using remimazolam rather than at 40–60, especially in elderly patients with cardiac dysfunction. In our case, both induction and maintenance doses of remimazolam may have led to an overdose because 1.0 mg of flumazenil was required for awakening. Flumazenil is a competitive antagonist of central benzodiazepine receptors [7]; therefore, the higher the residual concentration, the higher the dose required for antagonism. Because flumazenil has sympathomimetic effects owing to rapid arousal [8], its dosage should be minimized, particularly in elderly patients with cardiac dysfunction. Therefore, adjusting the intraoperative maintenance dose of remimazolam by observing BIS value and fluctuations in vital signs is necessary.

## 4. Conclusions

Here, we reported a case of postoperative HFpEF after antagonizing remimazolam with flumazenil. The maintenance dose of remimazolam is generally prone to resulting in overdose in elderly patients, in whom antagonism with flumazenil is necessary. To minimize the side effects of flumazenil, the maintenance dose of remimazolam in elderly patients with cardiac dysfunction should be customized according to their general condition.

## Abbreviations

HF: Heart failure
HFrEF: Heart failure with a reduced ejection fraction
HFpHF: Heart failure with a preserved ejection fraction
LVEF: Left ventricular ejection fraction
LA: Left atrium
BIS: Bispectral index
NT-proBNP: Amino-terminal pro-brain natriuretic peptide
E/A: Early to late diastolic transmitral flow velocity
BP: Blood pressure
EF: Ejection fraction.
